# Identification of QTN and candidate genes for Salinity Tolerance at the Germination and Seedling Stages in Rice by Genome-Wide Association Analyses

**DOI:** 10.1038/s41598-018-24946-3

**Published:** 2018-04-25

**Authors:** Shahzad Amir Naveed, Fan Zhang, Jian Zhang, Tian-Qing Zheng, Li-Jun Meng, Yun-Long Pang, Jian-Long Xu, Zhi-Kang Li

**Affiliations:** 1 Institute of Crop Sciences/National Key Facility for Crop Gene Resources and Genetic Improvement, Chinese Academy of Agricultural Sciences, 12 South Zhong-Guan-Cun Street, Beijing, China; 2 Shenzhen Institute of Breeding and Innovation, Chinese Academy of Agricultural Sciences, Shenzhen, China

## Abstract

To facilitate developing rice varieties tolerant to salt stress, a panel of 208 rice mini-core accessions collected from 25 countries were evaluated for 13 traits associated with salt tolerance (ST) at the germination and seedling stages. The rice panel showed tremendous variation for all measured ST traits and eight accessions showing high levels of ST at either and/or both the germination and seedling stages. Using 395,553 SNP markers covering ~372 Mb of the rice genome and multi-locus mixed linear models, 20 QTN associated with 11 ST traits were identified by GWAS, including 6 QTN affecting ST at the germination stage and 14 QTN for ST at the seedling stage. The integration of bioinformatic with haplotype analyses for the ST QTN lets us identify 22 candidate genes for nine important ST QTN (*qGR3, qSNK1*, *qSNK12*, *qSNC1*, *qSNC6*, *qRNK2*, *qSDW9a*, *qSST5* and *qSST9*). These candidate genes included three known ST genes (*SKC1*, *OsTZF1* and *OsEATB*) for QTN *qSNK1 qSST5* and *qSST9*. Candidate genes showed significant phenotypic differences in ST traits were detected between or among 2–4 major haplotypes. Thus, our results provided useful materials and genetic information for improving rice ST in future breeding and for molecular dissection of ST in rice.

## Introduction

Enormous environmental changes have brought negative impact on food crops in the world. Among these environmental factors, salinity is known to be able to inhibit plant growth and ultimately cause various degrees of crop yield losses^1,2^. As the most important staple food in Asia, a significant portion of rice crops are gworn along the coastal areas where rice paddy fields suffer various degrees of salinity. Salinity affects rice growth during all developmental stages from seed germination to reproduction^3^. With the development and spread of direct-seeding technology in many Asian countries, which requires high levels of germination and seedling establishment to achieve good harvests, salt tolerance (ST) at the seed germination and seedling stages has become a major rice breeding goal of these countries.

Most rice lines are sensitive to salt, particularly at the germination and seedling stage, but there is rich variability for ST among different rice accessions. It has been reported that some rice landraces such as Pokkali and Nona Bokra can withstand certain levels of salt stress^4^. Rice ST is a complex phenomenon trait both genetically and physiologically^5–8^. Thus, to efficiently exploit this genetic diversity of ST in breeding programs, it is important to identify quantitative trait loci (QTL)/quantitative trait nucleotides (QTN)/genes for ST at the seed germination and seedling stages so that marker-assisted breeding approach can be facilitated. Robin *et al*.^9^ used backcross population of IR62266-426-2 × IR60080-46A for mapping of osmotic related QTL. They identified 14 QTL on 8 chromosomes. Hossain *et al*.^10^ studied agronomic traits at the seedling and reproductive stages under saline condition and identified 16 QTL for salt tolerance, using an F_2_ population of Cheriviruppu × Pusa Basmati 1. Ghomi *et al*.^11^ identified 41 QTL affecting twelve physiological traits related to ST using 148 F_2_ population derived from Gharib (*indica*) × Sepidroud (*indica*). Sabouri *et al*.^12^ mapped 14 QTL in rice for physiological traits related to salt tolerance, using F_2_ population derived from Tarommahali and salt sensitive cv. Khazar.

In addition to genetic analyses of ST by QTL mapping, several QTL genes for rice ST have been cloned. These included *SKC1* which encodes a member of HKT-type transporters and is involved in maintaining K^+^ homeostasis in the ST variety under salt stress^13^, *OsCCC1* (cation-Cl^−^ cotransporter) that *OsCCC1* is reportedly involved in K^+^ and Cl^−^ transport and plays a significant role in K^+^ and Cl^−^ homeostasis and rice development^14^. *OsEATB* is also a cloned gene associated with tillering and panicle branching from rice variety 9311 which was down-regulated under salinity^15^. Other cloned ST genes included *OsMAPK33* whose over-expression under salinity increased Na^+^ uptake, suggesting its negative role in ST^16^, and *OsNHX1*, a Na^+^/H^+^ antiporter of rice^17^ whose expression in roots and shoots increases under high NaCl and KCl.

To date, large numbers of ST QTL have been mapped in rice, but most were identified by using bi-parental segregating populations involving a limited number of parents, which is unlikely to reveal the whole genetic variation for ST in rice germplasms^18^. Recently, natural population has been widely used in identifying QTL/QTN for complex traits, which have some advantages over the bi-parental populations^19,20^. Emon *et al*.^21^ used rice landraces and found 220 different markers associated with ST, eight of which were sequence-tagged-site markers developed for genes *SKC1*, *SalT* and *DST*. Using 220 rice accessions, Kumar *et al*.^22^ identified 20 SNPs significantly associated with Na^+^/K^+^ ratio, which explained 5–18% of the phenotypic variance. Negrão *et al*.^23^ targeted several markers involved in Na^+^/K^+^ ratio maintenance and found the transmembrane domain interaction of *OsHKT1;5* and *P140A* in ST using 392 rice lines. Platten *et al*.^24^ used 103 rice accessions for mining alleles at *OsHKT1; 5* locus and identified seven major and three minor alleles for Na^+^ in shoots at the seedling stage. Mishra *et al*.^25^ studied the association of natural genetic variations and haplotype distribution in rice for salt responsive candidate genes and found 22 salt responsive candidate genes for different ST phenotypes and haplotypes were identified in the Indian germplasms. Wang *et al*.^26^ used six flowering time related traits in *Arabidopsis thaliana* and improved the power of QTN detection in GWAS.

In this study, we used 208 diverse rice lines collected from 25 countries and 700 K high-density SNPs to conduct an association analysis to identify candidate genes and haplotypes associated with ST of rice and to reveal the genetic relationship between ST at the germination and seedling stages. The results provide valuable insights into the genetic basis of ST in rice that could be important for rice production and improvement.

## Materials and Methods

### Germplasms

A total of 219 germplasm accessions of rice from 25 different counties of Asia, Africa and Latin America were utilized in this study. The panel consists of landraces, commercial lines and advanced breeding lines, which showed tremendous phenotypic diversity for many agronomic traits^27^. FL478 and IR29 were used as the tolerant and sensitive checks, respectively^28,29^. For the nomenclature of the panel, accessions/lines of unknown country origin or with less than five samples from any single country were coded as CC, while the rest were coded with their respective country names, i.e. CH (China), Ind (India), Ban (Bangladesh), Phi (Philippines), Tha (Thailand), Tai (Taiwan) and Sri (Sri Lanka), etc. with their core collection serial numbers. As previously indicated^27^, two subgroups of the 219 associations were found by the 3-dimension principal component analysis (PCA) plot (Supplementary Fig. S1). Given the strong population differentiation of the panel, 11 accessions were removed and the remaining 208 accessions showing less pronounced population differentiation were used for the following analysis in this study.

### Evaluation of ST at the germination stage

Ten healthy seeds of each accession were selected and placed on to two filter papers soaked with 10 ml 100 mM NaCl in a petri plate for screening ST at the germination stage. For the controlled experiment, the same number of seeds of each line was placed on the filter papers soaked with 10 ml distilled water in a petri plate. All petri plates were incubated under the controlled conditions in the growth chamber with 25 ± 2 °C temperature, a 12-h light period and 60% moisture. The petri dishes were arranged in a completely randomized design with two replications for each accession. Seed sprouting and germination were recorded on daily basis. At 10 days after soaking, the final germination rate (GR) was calculated for each genotype^30^. Root length (RL) and seedling length (SL) were measured for all geminated seeds of each genotype on the 10^th^ day after soaking.

### Evaluation of ST at the seedling stage

The experiment for evaluating ST at the seedling stage was carried out in the glasshouse facility at Institute of Crop Sciences, the Chinese Academy of Agricultural Sciences (CAAS) in Beijing. The glasshouse conditions were set as 30–20 °C (day-night) and 60–65% relative humidity were regulated with a roof net, ventilation system and cooling pad. Plastic containers were prepared for the screening purpose and styrofoam sheets were used to fit inside of each container. Each styrofoam contains 10 × 13 holes, at the bottom of each styrofoam nylon net was stitched to prevent seeds from falling into the solution^31^. The seeds were surface sterilized with 5% fungicide (sodium hypochlorite) solution for 20 minutes and rinsed well with distilled water. Seeds were soaked for 48 h and one healthy germinated seed was placed in per hole, ten holes were used for per genotype. To recover the radical and plumule, normal tap water with pH 5 was used in the containers for two days. At the 3^rd^ day, saline solution with 70 mM and nutrients were applied, which is 50% of the actual stress. At the 6^th^ day, the saline solution was raised to 140 mM and nutrients were used in the containers^32^. Nutrients solution pH (5.1 to 5.5) and glasshouse conditions were maintained on a regular basis. The solutions were changed every 5th day. When the susceptible check IR29 showed complete susceptibility to salinization, score for salt toxicity symptoms (SST) was evaluated according to the modified Standard Evaluation System (SES)^33^. Seedling survival days (SSD) were recorded on daily basis since first plant died. Finally, at the 16^th^ day after salinization, whole plant samples of each accession were collected, dried, and then measured for root dry weight (RDW) and shoot dry weight (SDW) using a High Precision Digital Balance.

### Measurement of Na^+^ and K^+^ concentrations

A separate second experiment was conducted to measure physiological traits related to ST, such as Na^+^ and K^+^ concentrations in the shoots and roots. The screening protocol of the second experiment was similar to the first experiment. The germinated seeds of 208 lines were sown in separate 96 wells PCR plates, with perforate wells at the bottom to facilitate the roots to have contact with the saline solution. Three replications were performed to minimize environmental errors. The shoots and roots were harvested and rinsed with distilled water several times after 8 d of salinization with 140 mM of NaCl. Samples were dried for 72 h at 60 °C in Blue Pard Oven (DHG-9240A), then weighed, and extracted in acetic acid (100 mM L^−1^) at 90 °C for 2 h. Root samples were diluted with distilled water as 1:11 for root Na^+^ concentration (RNC) and 1:2 for root K^+^ concentration (RKC) determination and shoot samples were diluted with distilled water as 1:24 for shoot Na^+^ concentration (SNC) and shoot K^+^ concentration (SKC) determination. Two replicates were performed per sample and the average value of the replicates was taken. Ratio of Na^+^ and K^+^ concentrations in shoots to roots (SNK and RNK) were determined by dividing the respective values of SNC and RNC by SKC and RKC, respectively. Sodium and potassium were determined by the atomic absorption spectrophotometer (S2, Solar House, United Kingdom). Concentrations of sodium and potassium in shoots and roots were expressed in millimoles per gram (mM g^−1^).

### Genotyping dataset

High-density rice array (HDRA) comprised of 700 K SNPs were used for genotyping the panel. The HDRA was developed as an Affymetrix Custom Gene Chip Array from a SNP discovery dataset of about ~16 M SNPs (generated by re-sequencing 128 rice samples at ~7X genome coverage)^34^. SNPs with minor allele frequency (MAF) less than 0.05 were culled and finally 395,553 SNPs were used for GWAS.

### Genome-wide association studies

According to seedling survival days and corresponding number of survival plants every day, weighted average of SSD at the 16^th^ day after salinization was calculated and used for data analysis. Pearson’s correlations among the phenotypic traits measured at the germination and seedling stages were calculated by using SAS PROC CORR^35^. Analyses of variance was performed to determine significances of variation due to replications and genotypes for all measured ST traits by SAS PROC GLM^35^.

For QTN identification, 395,553 SNPs and 208 accessions were used to detect SNP-trait associations using GWAS. Marker-trait associations were conducted by compressed mixed linear model implemented in GAPIT with the default settings except for the PCA setting as 3^36^. A threshold of *p*-value (1.0 × 10^−5^) was used to claim significant SNP-trait associations and significant SNPs in <200 kb distances were considered as a single QTN^37,38^.

The multi-locus GWAS analysis was performed by mrMLM package (https://cran.r-project.org/web/packages/mrMLM/index.html) in R software and critical LOD score for significant QTN were used for the confirmation of the identified QTN^26^.

### Important QTN and candidate gene identification

For selecting candidate genes in important QTN regions, QTN meeting at least one of the following criteria were considered as important: (1) close (<1 Mb) to the cloned genes or fine mapped QTL; (2) accounting for over 10% of the phenotypic variance^37,38^. The following three steps were conducted to identify candidate genes for each important QTN: (I) identify all non-synonymous SNPs in CDS of all genes located inside the important QTN region; (II) genes containing SNPs detected with −log_10_(*p*) >3; and (III) statistically significant differences detected between different major haplotypes (containing more than 8 samples) consisting of all non-synonymous SNPs within each of previous identified candidate genes within the QTN region.

## Results

### Phenotypic variation and trait correlations

Considerable phenotypic variations were observed for all 13 ST traits measured in the current rice panel at both the germination and seedling stages (Table 1). ANOVA results showed that differences among the genotypes explained an average 84% of the phenotypic variance for the traits at the seedling stage, ranging from 51.3% for shoot dry weight (SDW) to 96.7% for ratio of Na^+^ and K^+^ concentrations in roots (RNK) (Supplementary Table S1). Among the 208 accessions, ten lines (Ind143, Ind136, Ind110, Ind195, Ban108, CH29, Sri266, CC279, CC274 and CC6) showed the same high level of ST as FL478 (the ST check) with the lowest average score for salt toxicity symptoms (SST) of 4 and seedling survival days (SSD) of more than 12 d. Similarly, differences among genotypes explained an average of 88.3% of the total phenotypic variance for ST traits measured at the germination stage, ranging from 82.4% for germination rate (GR) to 95.2% for seedling length (SL). While the 208 accessions had an average GR of 29.4%, (Table 1), 6 lines (Phi4, Phi10, CC66, Tha212, CH206, and Tai260) showed very high GR >75% with significantly longer roots and shoots than the tolerant check under the 100 mM NaCl salt stress.

**Table 1:** Performances of salt tolerance related traits measured at germination and seedling stages of the 208 rice germplasms.

Trait^1^	Mean ± SD	Range	CV (%)
RL(cm)	0.90 ± 0.21	0.10–2.30	26.6
SL (cm)	2.46 ± 0.43	0.80–4.20	21.7
GR (%)	29.4 ± 9.5	0.0–85.0	36.1
RDW (g)	0.02 ± 0.00	0.02–0.06	22.1
SDW (g)	0.10 ± 0.00	0.06–0.17	21.2
SST	7.42 ± 1,27	3.00–9.00	16.1
SSD (d)	9.42 ± 0.60	7.50–13.10	9.3
RNC (mM)	1.32 ± 0.20	0.38–1.99	27.1
SNC (mM)	2.12 ± 0.46	0.49–3.92	26.2
RKC (mM)	0.41 ± 0.00	0.07–1.01	26.7
SKC (mM)	0.53 ± 0.3	0.12–1.21	29.1
RNK	2.31 ± 1.35	0.09–3.92	28.5
SNK	2.95 ± 0.96	0.94–3.80	29.8

^1^RL: root length, SL: seedling length, GR: germination rate, RDW: root dry weight, SDW: seedling dry weight, SST: score for salt toxicity symptoms, SSD: seedling survival days, RNC: root Na^+^ concentration, SNC: shoot Na^+^ concentration, RKC: root K^+^ concentration; SKC: shoot K^+^ concentration, RNK: ratio of Na^+^ to K^+^ concentrations in roots; SNK: ratio of Na^+^ to K^+^ concentrations in shoots.

Significant and positive correlation was observed between root length (RL), SL and GR at the germination stage (Table 2). At the seedling stage, only the shoot Na^+^/K^+^ ratio (SNK) was significantly correlated with the final ST traits, SST and SSD. Significant and positive correlation was observed only between shoot Na^+^ concentration (SNC) and root K^+^ concentration (RKC) and between SST and shoot Na^+^/K^+^ ratio (SNK). Significant negative correlation was observed between SST and SSD, and between SNC and shoot K^+^ concentration (SKC). No correlation was observed between the ST traits measured at the germination stage and those at the seedling stage, except for a weak but significant negative correlation between SL at the germination stage and SNK at the seedling stage, indicating ST at the two developmental stages of rice were largely under independent genetic controls.

**Table 2:** Pearson’s co-efficient of correlation for salt tolerance related traits measured at the germination and seedling stages under salinity.

Sr#	Trait^1^	RL	SL	GR	RDW	SDW	SST	SSD	RNC	SNC	RKC	SKC	RNK
1	SL	0.57**											
2	GR	0.80**	0.54**										
3	RDW	0.04	0.01	0.08									
4	SDW	0.12	0.07	0.14	0.09								
5	SST	0.02	−0.06	0.01	−0.05	−0.25**							
6	SSD	0.05	0.15	0.06	0.08	0.27**	−0.79**						
7	RNC	−0.06	0.09	−0.01	−0.25**	−0.12	0.05	−0.01					
8	SNC	0.07	0.08	0.05	−0.04	−0.27**	−0.01	0.08	−0.01				
9	RKC	−0.11	0.06	0.17	−0.13	0.05	−0.28**	0.31**	0.53**	0.01			
10	SKC	0.06	0.11	0.05	−0.02	−0.13	−0.21*	0.26**	−0.03	−0.82**	0.054		
11	RNK	0.00	0.11	0.09	−0.07	−0.12	0.24**	−0.29**	0.35**	−0.05	−0.50**	−0.115	
12	SNK	−0.07	−0.20*	−0.11	0.001	−0.27**	0.48**	−0.54**	0.12	0.01	−0.19*	−0.28**	0.27**

^1^Same as in Table 1.

* and ** represents significance at P < 0.05 and P < 0.01, respectively.

### SNP markers

**Figure 1:**
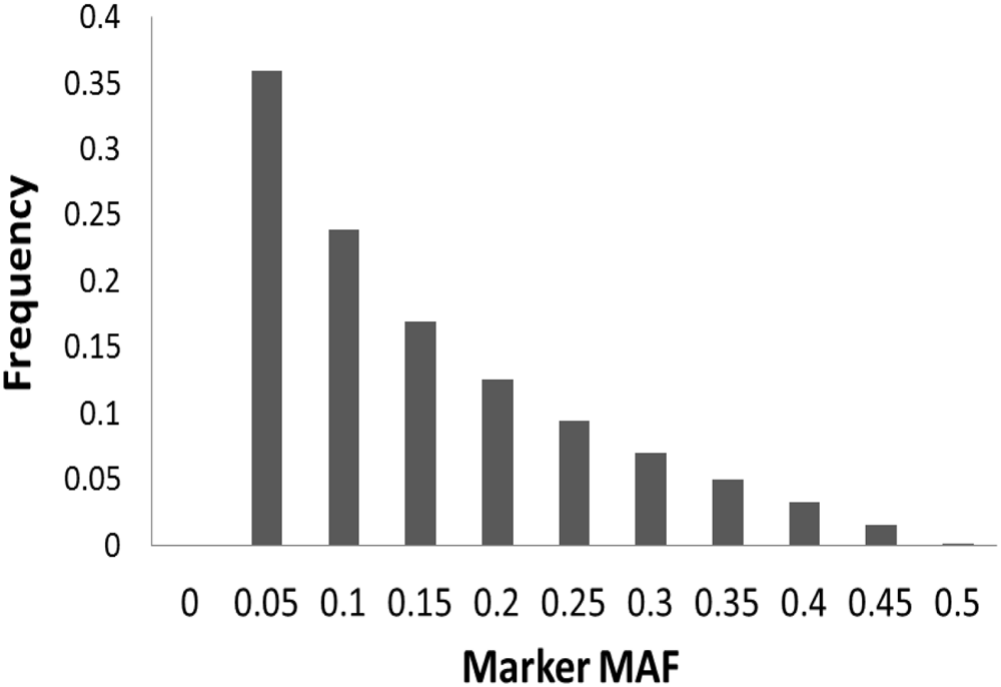
Frequency of markers in different MAF Classes.

In total, 395,553 SNPs covering 372 Mb of the rice genome were used for genetic analysis of the population. SNPs were well distributed throughout the genome, ranking from 25,045 SNPs (Table 3) covering 22.90 Mb of chromosome 9 to 47,896 SNPs spanning 43.25 Mb of chromosome 1 with an average distance of ~1 kb between adjacent SNPs. Relative large gaps of 107, 112, 114, 124, 238 and 528 kb between adjacent SNPs were observed on chromosomes 2, 12, 10, 7, 4 and 11, respectively (Table 2). Among SNPs with minor allele frequency (MAF) ≥0.05, there was about 51% of the markers having MAF lower than 0.1 (Fig. 1).

**Table 3:** Genome wide basic statistics of the SNP markers among the 208 rice germplasm accessions.

Chr	Size (Mb)	No. of SNPs	Coverage (%)	Average gap (kb)	Largest Gap (kb)
1	43.25	47,896	96.0	0.90	58
2	35.93	40,369	97.6	0.89	107
3	36.39	36,752	97.7	0.99	33
4	35.46	35,119	98.9	1.00	238
5	29.90	28,729	99.5	1.04	75
6	31.24	31,993	97.3	0.97	76
7	29.69	28,919	97.8	1.02	124
8	28.44	30,496	99.7	0.93	95
9	22.90	25,045	96.1	0.91	85
10	23.20	23,531	98.1	0.98	114
11	29.02	36,842	94.1	0.78	528
12	27.53	29,863	99.2	0.92	112
Total	372.96	39,5553	97.7

### QTN for ST at the germination stage

In total, six QTN affecting the three traits related to rice ST at the germination stage were detected (Table 4). Four QTN (*qSL2*, *qSL6, qGR4* and *qRL8*) were identified using GAPIT and confirmed by mrMLM while two additional QTN (*qGR3* and *qRL12*) were detected by mrMLM. For SL, two QTN (*qSL2* and *qSL6*) were identified on chromosomes 2 and 6 with LOD values of 6.06 and 8.08, which explained 9.6% and 9.2% of the total SL phenotypic variance, respectively. The estimated genetic effect was 0.28 for *qSL2* and 0.19 for *qSL6*. For GR, two QTN (*qGR3* and *qGR4*) were identified on chromosomes 3 and 4 with LOD values of 4.31 and 3.02, which accounted for 9.6 and 9.1% of the total phenotypic variance and had fairly large genetic effects of 7.69 and 8.49 in germination rate. For RL, two QTN *qRL8* and *qRL12* were identified on chromosomes 8 and 12, which accounted for 9.7 and 8.5% of the phenotypic variance with genetic effects of 0.23 and 0.77 in RL, respectively.

**Table 4:** QTN identified for salt tolerance related traits measured at germination and seedling stages.

Trait^1^	GWAS by compressed mixed linear model implemented in GAPIT									The multi-locus GWAS confirmed by mrMLM^2^	Comparative mapping with the previously reported Gene/QTN^3^
	QTN	Chr	Peak SNP	Range (Mb)	LOD score	MAF	R^2^ (%)	Allele	Effect		
SL	*qSL2*	2	9675710	9.60–9.80	6.06	0.11	9.6	G/A	0.28	a	*OsGMST1* ^43^
	*qSL6*	6	8516988	8.51–8.71	8.08	0.1	9.2	G/C	0.19	a	
GR	*qGR3*	3	6884762	6.83–7.19	4.31	0.12	9.6	G/A	7.69	b	*DSM3* ^44^
	*qGR4*	4	4660530	4.50–4.70	3.02	0.28	9.1	A/G	8.49	a	
RL	*qRL8*	8	12602143	12.50–12.70	2.85	0.1	9.7	G/A	0.23	a	*OsCCC1* ^14^
	*qRL12*	12	22562394	22.55–22.68	3.05	0.09	8.5	G/A	0.77	b	
SKC	*qSKC9*	9	6625407	6.60–6.80	3.89	0.12	10.8	C/G	10.09	a	
	*qSKC11*	11	22720171	22.91–23.11	5.22	0.12	9.1	T/C	15.28	a	
RNK	*qRNK2*	2	24249696	24.24–24.40	6.04	0.07	10.7	G/T	0.34	a	*RSS1* ^45^
SNK	*qSNK1*	1	11506233	11.40–11.60	4.99	0.24	10.3	G/T	−0.59	a	*SKC1* ^13^
	*qSNK12*	12	4055569	3.94–4.10	5.99	0.34	10.1	C/G	0.33	a	
SNC	*qSNC1*	1	23751820	23.75–23.94	4.16	0.15	10.1	C/G	20.98	a	
	*qSNC6*	6	17675871	17.50–17.70	9.01	0.82	10.4	G/C	31.52	a	
RDW	*qRDW10*	10	14798892	14.71–14.90	4.41	0.17	8.9	C/T	0.1	a	
SDW	*qSDW9a*	9	1770818	1.70–1.90	6.92	0.21	10.7	A/G	0.13	a	
	*qSDW9b*	9	4460376	4.42–4.62	4.31	0.24	10.1	T/C	0.13	a	
SST	*qSST5*	5	7014412	7.00–7.20	4.13	0.09	10.4	G/A	−0.69	a	*OsTZF1* ^46^
	*qSST6*	6	8419190	8.40–8.60	6.1	0.43	9.6	G/A	−0.47	a	
	*qSST9*	9	20746981	20.61–20.79	4.88	0.07	10.7	C/T	−1.08	a	*OsEATB* ^15^
SSD	*qSSD5*	5	7014412	7.01–7.17	4.72	0.08	10.8	G/A	0.37	a	

^1^Same as in Table 1.

^2^a = loci confirmed by mrMLM, b = additional loci detected by mrMLM.

^3^Numbers on top right corner of genes are serial number of references.

### QTN for ST at the seedling stage

In total, 14 QTN were identified for eight seedling stage ST related traits. These QTN locate on all rice chromosomes except 3 and 7 and explained 8.9–10.8% of the trait phenotypic variances (Table 4).

For SKC, two QTN (*qSKC9* and *qSKC11*) were identified on chromosomes 9 and 11 with LOD value 3.89 and 5.22 and explained 10.8% and 9.1% of the trait phenotypic variance, respectively, which had genetic effects of 10.1 and 15.3 in SKC. For RNK, one QTN (*qRNK2*) was identified on chromosome 2 with LOD of 6.04, explaining 10.7% phenotypic variance with a genetic effect of 0.34 in RNK. For SNK, two QTN (*qSNK1* and *qSNK12*) were identified on chromosomes 1 and 12 with LOD value 4.99 and 5.99 and explained 10.3% and 10.1% of the trait phenotypic variance with genetic effects of −0.59 and 0.33, respectively in SNK. For SNC, two QTN, *qSNC1* and *qSNC6*, were identified on chromosomes 1 and 6 with LOD value 4.16 and 9.01 and accounted for 10.1% and 10.4% of the phenotypic variance, respectively. For RDW, one QTN (*qRDW10*) was identified on chromosome 10 with LOD values of 4.41 and explained 8.9% of phenotypic variance. For SDW, two QTN (*qSDW9a* and *qSDW9b*) were identified on chromosome 9 with LOD values of 6.92 and 4.31, which accounted for 10.7% and 11.1% of phenotypic variance, respectively. For SST, three QTN (*qSST5*, *qSST6* and *qSST9*) were identified on chromosomes 5, 6 and 9 with LOD values of 4.13, 6.10 and 6.88, which explained phenotypic variance of 10.4%, 9.6% and 10.7%, respectively. For SSD, one single QTN (*qSSD5*) was identified on chromosome 5 with a LOD value of 4.72 and explained 10.8% of phenotypic variance. Surprisingly, none of the above QTN were mapped closely one to another.

### Candidate genes for important QTN

With the resolution of 200 kb for all identified QTN, we were able to narrow down a relatively small number of candidate genes for each of the identified QTN. We focused on those candidate genes that locate within the 200 kb region of each identified QTN and also contain nonsynonymous SNPs in their CDS regions among the 208 rice panel. We found three cloned ST genes, *OsGMST1, DSM3* and *OsCCC1*, locate within the vicinities (0.00–1.3 Mb) of three QTN (*SL2, qGR3* and *qRL8*) identified at the germination stage. Significant (−log_10_(*P*) >3) non-synonymous SNPs were identified in the CDS region of *DSM3*, but not in the CDS regions of *OsGMST1* and *OsCCC1*.

**Figure 2:**
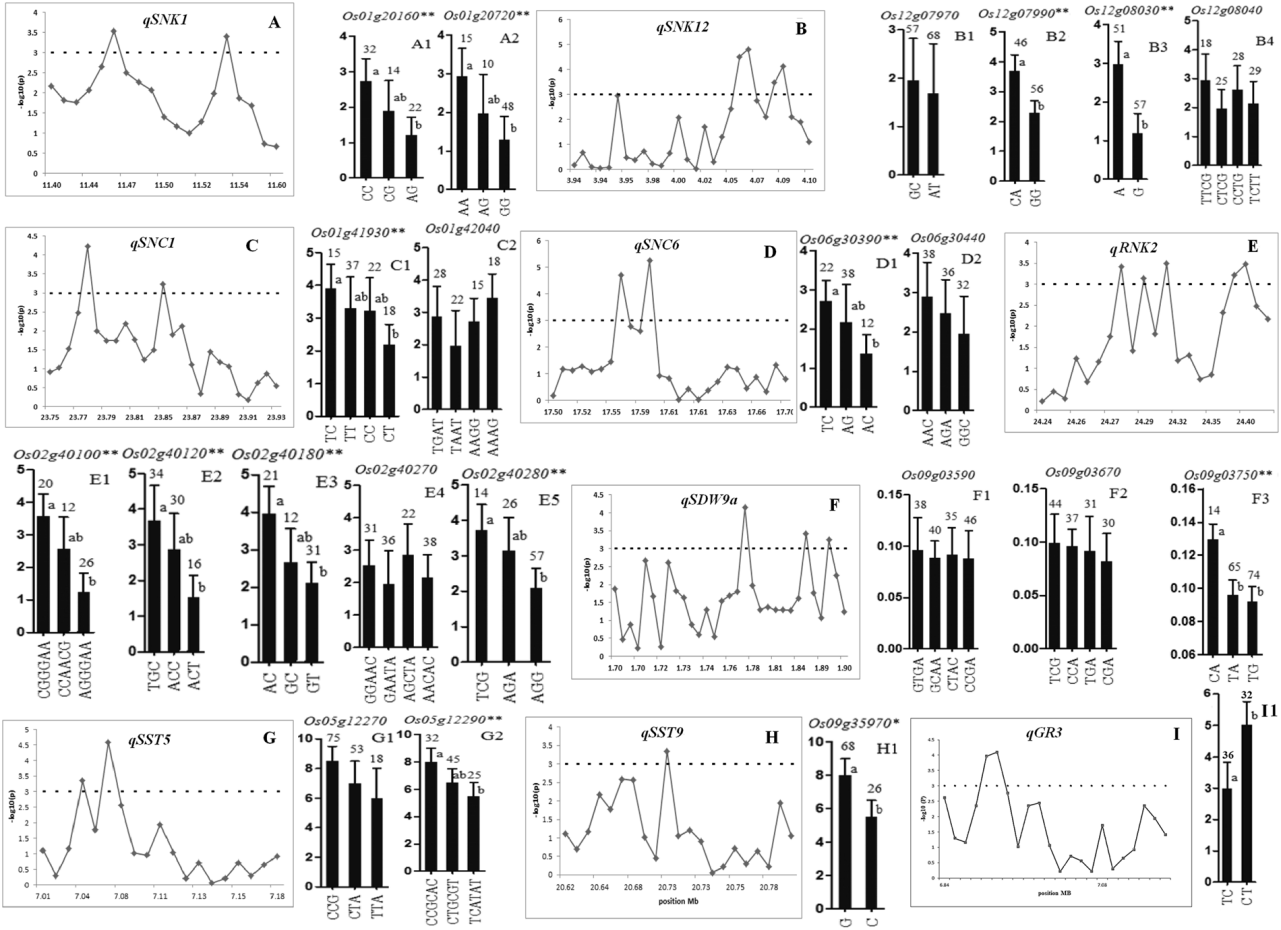
Manhattan plot of important QTN and haplotype analysis of candidate genes. These genes are related to corresponding QTN including: analysis of candidate genes was done by using SAS general linear model approach *qSNK1* (**A**), *qSNK12* (**B**)*, qSNC1* (**C**)*, qSNC6* (**D**)*, qRNK2* (**E**), *qSDW9a* (**F**) *qSST5* (**G**), *qSST9* (**H**) and *qGR3* (**I**). Each point was a gene in 200 kb region of the peak SNP of QTN. Dot line showed the threshold to determine candidate genes. The * and ** suggested significance of ANOVA at P < 0.05 and 0.01. The letter on histogram (a and b) indicated multiple comparisons result at the significant levels 0.05 and 0.01. The value on top of histogram was the number of individuals for each haplotype and histogram length shows the mean value of these individuals for each haplotype

For those QTN associated with ST traits at the seedling stage, 22 candidate genes for nine important QTN (*qGR3, qSNK1, qSNK12, qSNC1, qSNC6, qRNK2, qSDW9a, qSST5* and *qSST9*) were identified based on the two criteria (Table 5, Supplementary Table S2). Significant phenotypic differences were detected between haplotypes at 13 of these candidate genes (Fig. 2, Supplementary Table S3). For *qSNK1* mapped to a confidence interval of 11.40–11.60 Mb on chromosome 1, we detected 107 SNPs inside 19 genes with two most significant candidates, *Os01g20160* and *Os01g20720*. Three haplotypes were identified for each of the two candidate genes and significant phenotypic differences among them were observed by ANOVA (Fig. 2, A1-A2). For *qSNK12*, the confidence interval of 3.94–4.10 Mb on chromosome 12 included 130 SNPs located inside 28 genes and four significant candidate genes (*Os12g07970, Os12g07990, Os12g08030* and *Os12g08040*) were identified with −log_10_(*P*) >3. Two haplotypes were identified for each of the two candidate genes (*Os12g07990* and *Os12g08030*) with significant phenotypic differences detected between the two haplotypes at each of the two candidates (Fig. 2, B1-B4). The confidence interval of *qSNC1* in 23.75–23.94 Mb on chromosome 1 covered 152 SNPs inside 25 genes with two significant candidate genes (*Os01g41930* and *Os01g42040*) identified with −log_10_(*P*) >3. Significant phenotypic differences were detected among four haplotypes at *Os01g41930* (Fig. 2, C1-C2), suggesting it is the most likely candidate for *qSNC1*.

**Table 5:** List of 22 candidate genes for seven important QTN identified at seedling stage under salinity.

QTN	MSU ID	Annotation
*qSNK1*	*Os01g20160*	*OsHKT1*;5 - Na^+^ transporter, expressed
*qSNK1*	*Os01g20720*	CC-NBS-LRR, putative, expressed
*qSNK12*	*Os12g07970*	Transporter, major facilitator family, putative, expressed
*qSNK12*	*Os12g07990*	Protein kinase family protein, putative, expressed
*qSNK12*	*Os12g08030*	Expressed protein
*qSNK12*	*Os12g08040*	Retrotransposon protein, putative, unclassified
*qSNC1*	*Os01g41930*	Leucine rich repeat protein, putative, expressed
*qSNC1*	*Os01g42040*	Ubiquitin-conjugating enzyme, putative, expressed
*qSNC6*	*Os06g30390*	Oxidoreductase/transition metal ion binding protein, putative, expressed
*qSNC6*	*Os06g30440*	*OsGH3*.7-Probable indole-3-acetic acid-amidosynthetase, expressed
*qRNK2*	*Os02g40100*	Plant protein of unknown function *DUF869* domain containing protein, expressed
*qRNK2*	*Os02g40120*	Expressed protein
*qRNK2*	*Os02g40180*	Receptor-like protein kinase 5 precursor, putative, expressed
*qRNK2*	*Os02g40270*	Expressed protein
*qRNK2*	*Os02g40280*	piwi domain containing protein, putative, expressed
*qSDW9a*	*Os09g03590*	Retrotransposon protein, putative, unclassified, expressed
*qSDW9a*	*Os09g03670*	Retrotransposon protein, putative, unclassified, expressed
*qSDW9a*	*Os09g03750*	Ankyrin, putative, expressed
*qSST5*	*Os05g12270*	Expressed protein
*qSST5*	*Os05g12290*	Retrotransposon protein, putative, unclassified, expressed
*qSST9*	*Os09g35970*	TBC domain containing protein, expressed
*qGR3*	*Os03g12840*	Inositol 1,3,4-trisphosphate 5/6-kinase, putative, expressed

The confidence interval 17.50–19.70 Mb of *qSNC6* on chromosome 6 contains 100 SNPs within 25 genes and two candidate genes (*Os06g30390* and *Os06g30440*) were highly significant with −log_10_(*P*) >3. Significant phenotypic differences in SNC were detected among the three haplotypes at *Os06g30390* (Fig. 2, D1-D2), suggesting it is the most likely candidate gene for *qSNC6*. The *qRNK2* confidence interval of 24.24–24.40 Mb on chromosome 2 contains 137 SNPs within 21 genes with five significant ones (*Os02g40100*, *Os02g40120*, *Os02g40180*, *Os02g40270* and *Os02g40280*) with −log_10_(*P*) >3 as the likely candidates. We identified three haplotypes at four candidate genes (*Os02g40100, Os02g40120, Os02g40180* and *Os02g40280*) with significant phenotypic differences among them for RNK (Fig. 2, E1-E5). The *qSDW9a* confidence interval of 1.70–1.90 Mb on chromosome9 contains170 SNPs within 28 genes and three genes (*Os09g03590, Os09g03670* and *Os09g03750*) were most likely candidate genes with −log_10_(*P*) >3. We detected significant phenotypic differences for SDW among the three haplotypes at *Os09g03750* (Fig. 2, F1-F3), suggesting *Os09g03750* was the most likely candidate for *qSDW9a*.

There are 62 SNPs within 20 genes in the *qSST5* confidence interval 7.0–7.2 Mb on chromosome 5. Two of these genes, *Os05g12270* and *Os05g12290*, were the more likely candidate genes because of statistical significance at −log_10_(*P*) >3. We detected three haplotypes with significant difference for SST among them (Fig. 2, G1-G2).

The *qSST9* confidence interval 20.61–20.79 Mb on chromosome 9 contained 102 SNPs within 20 genes with *Os09g35970* showing the highest level of significance and thus considered as the most likely candidate. Highly significant phenotypic differences for SST were detected between two haplotypes at *Os09g35970* in the rice panel (Fig. 2, H1). For *qGR3* mapped to a confidence interval of 6.83–7.19 Mb on chromosome 3, we detected 63 SNPs inside 19 genes with one most significant candidates, *Os03g12840*. Two haplotypes were identified and significant phenotypic differences between them were revealed by ANOVA (Fig. 2, I1).

## Discussion

### Genetic diversity of ST traits at the germination and seedling stages in rice

In the coastal areas of south and southeast Asian countries where salinity is a major threat limiting rice productivity, progress in improving ST of rice by breeding would have significant impact in the food security and poverty alleviation of these countries, particularly when facing the rising sea level from the global warming. However, developing ST varieties requires sufficient genetic diversity for ST within the primary gene pool of rice. Consistent with previous reports^39^, we observed the tremendous genetic diversity for all measured ST traits in the 208 accessions evaluated and identified eight accessions of diverse origins that showed high levels of ST at either and/or germination and seedling stages. These lines can be used directly as donors of ST in rice breeding programs for genetic improvement of ST or as excellent materials for genetic and molecular dissection of ST in rice. The absence of phenotypic correlation between the ST traits at the germination stage and those at the seedling stage was consistent with previous reports^30,40^. This was expected from the fact that most genes affecting rice abiotic stress tolerances such as ST and drought tolerance are developmentally regulated^41–43^. This result would imply that developing rice varieties with greatly improved ST at both the germination and seedling stages can be achieved in breeding, but phenotypic screen of segregating breeding populations for ST should be carried out at different developmental stages when breeding is aiming at improving ST at multiple developmental stages.

### QTN and candidate gene identification for ST traits by GWAS

In this study, identification of many QTN and their candidate genes/alleles for ST traits of rice by GWAS should be considered more efficient when compared with most QTN mapping studies of similar scale by linkage mapping^11,12^. Out of the 20 ST QTN identified, seven were found to locate in the same loci or adjacent to the cloned ST genes reported previously (Table 4). For example, *qSL2* (9.60–9.80 Mb on chromosome 2) associated with SL at the germination stage was adjacent to the cloned ST gene *OsGMST1*, which is specifically expressed under salt stress, while reduced expression of this gene is associated with hypersensitivity to salt stress in rice^44,45^. Similarly, *qGR3* (6.83–7.19 Mb on chromosome 3) associated with GR at the germination was adjacent to *DSM3*, which is an important member of the *OsITPK* family. *DSM3* produces stress resistance under drought and salt in rice^46^. *qRL8* (12.50–12.70 Mb on chromosome 8) associated with RL at the germination under salinity was adjacent to the cloned ST gene, *OsCCC1* (cation-Cl^−^ cotransporter)^14^, which is involved in K^+^ and Cl^−^ transport and had a significant role in K^+^ and Cl^−^ homeostasis and rice plant development. *qRNK2* in the region of 24.24–24.40 Mb on chromosome 2 was mapped in a genomic region adjacent to *RSS1* that is reportedly able to maintain the vigor and viability of the meristematic cells under salinity, while inadequate functioning of *RSS1* would cause an extreme dwarf and short-root phenotype under high-salt but not under the normal growth conditions^47^. Others included *qSNK1* in the region of 11.40–11.60 Mb on chromosome 1, which affected SNK in shoots, was mapped in the genomic region adjacent to the cloned ST gene, *SKC1* encoding a member of HKT-type transporters for K^+^ homeostasis in rice shoots^13^. *qSST5* was mapped in the adjacent region of *OsTZF1*, a member of the CCCH-type zinc-finger gene family of rice whose expression induces ST and drought tolerance in rice^48^. *qSST9* associated SST in the region of 20.61–20.79 Mb on chromosome 9 was mapped together with the cloned gene *OsEATB*^15^, which was reportedly down-regulated and associated with tillering and panicle branching under salinity. These results implied that the false positive probability would be very low for the ST QTN identified in this study.

Another major advantage in our QTN mapping by GWAS was the high resolution for the identified QTN. In this study, we used 395,553 SNPs in our genotyping and for QTN identification, which allowed us to narrow down many identified ST QTN each to a small genomic region <200 kb and shortlist candidate genes to a small number of genes for each of the identified QTN. Further, SNPs in CDS regions within each identified candidate genes were used for haplotype analyses for conforming the identified candidate genes, which allowed us to further shortlist very few or even a single candidate gene for some of the ST QTN. Using this strategy, we were able to shortlist 22 candidate genes for eight ST QTN identified in this experiment. These included two candidate genes, *Os01g20160* and *Os01g20720* for *qSNK1*. *Os01g20160* encodes an *OsHKT1*-5-Na^+^ transporter which was reportedly involved in Na^+^ transportation and K^+^ homeostasis^13^. *Os01g20720* encodes CC-NBS-LRR protein, which is known to be able to regulate the signaling pathway to establish rice defense mechanism under stress conditions^49,50^. Similarly, the most likely candidate genes for *qSNK12* included *Os12g07970* and *Os12g07990*. The former encodes a transporter and major facilitator family protein which the latter encodes putative protein of kinase family. Both proteins are reportedly involved in plant defense and stress tolerance^51^ and hence are the most likely candidate genes of *qSNK12*. Similarly, our result suggested *Os01g41930* and *Os01g42040* were the most likely candidates for *qSNC1*. *Os01g41930* encodes a leucine rich repeat protein which is up-regulated under cold and drought stress in rice^52^ while the result of haplotype analysis (Fig. 2, C1) suggested *Os01g41930* was most likely candidate gene for *qSNC1*. For *qSNC6*, *Os06g30390* was most likely candidate because it encodes an oxidoreductase protein expressed in plastids and is able to catalyze the oxidation and reduction process, thus is associated with salt susceptibility by indirectly neutralizing with other ions and avoiding the crystal formation with Na^+^ bonding^53^. The most likely candidate genes for *qRNK2* were *Os02g40100* and *Os02g40280* because the former encodes the *DUF869* domain containing protein, which was reportedly associated with root traits under drought conditions^54^ and thus potentially able to contribute to ST. The latter, *Os02g40280*, encodes a piwi domain protein which belongs to the argonaute protein family that are commonly induced under abiotic and viral stress in many plant species^55^. Similarly, our data suggested that *Os09g03750* was the most likely candidate for *qSDW9a* because it encodes an ankyrin (ANK) putative protein. ANK proteins are mainly involve in specific protein-protein interaction^56^. For *qGR3*, *Os03g12840* was most likely candidate gene for germination, it encodes for the production of putative inositol 1,3,4-trisphosphate 5/6-kinase and cause less accumulation of osmolytes and soluble sugar in root^46^. Currently, all the 22 candidate genes for rice ST are being verified by genetic transformation and further molecular analyses.

Consistent with previous reports^37,57^, our results demonstrated several advantages of GWAS such as high genetic diversity and high mapping resolution. We also, however, noted a few limitations of this approach. These included poor ability to detect rare QTN/QTL alleles and QTN of small effect and inability to detect epistasis. During our analyses, we tentatively removed the minor alleles to minimize the false positive probability, thus QTN associated with the unique or rare alleles were undetectable in this study. We also realized that in our haplotype analyses, uses of non-synonymous SNPs in gene CDS regions could only capture part of the phenotypic variation associated by those mutations, but unable to detect those mutations occurring in the gene promoter regions that are known to be able to change gene expression and cause trait variation. Because SNPs in the promoter regions of the candidate genes were not covered in our haplotype analyses, some of the QTN candidate genes could have been incorrectly predicted or missed. The most likely examples in this study included *qSL2* and q*RL8* for which we were not able to identify any significant non-synonymous SNPs in their QTN confidence intervals. An additional problem for the accuracy in identifying QTN and candidate genes by GWAS is expected to be affected to a certain degree of the gene presence and absence variation when a single reference genome is used in SNP calling and candidate gene prediction, because it is already known that a significant level of gene presence and absence variation exists in rice germplasm^58^. Fortunately, this problem can be largely overcome when more and more new reference genomes are publicly available^59^. Finally, with so many ST QTN and their candidate genes identified and the availability of high density SNP markers, it remains a big challenge to pyramid many ST QTN/genes for improving rice ST in breeding, even with the current genome selection technology^49,60,61^. The good news is that strong phenotypic selection under appropriate salt stress is able to pyramid and quickly fix large numbers of ST loci in very few generations^62^, even though this phenomenon remains poorly understood genetically and at the molecular level.

## Conclusion

Tremendous amounts of genetic diversity for 13 ST-related traits existed in rice germplasm. Using GWAS, 6 and 14 QTN were identified for ST-related traits at the germination and seedling stages, respectively, several of which were mapped to the genomic regions of previously cloned ST genes, including *SKC1* encoding a member of HKT-type transporters for K^+^ homeostasis, *OsTZF1* (a member of the CCCH-type zinc-finger gene family) for ST and drought tolerance in rice and *OsEATB* associated with tillering and panicle branching under salinity. A total of 22 candidate genes and important ST alleles were identified for nine important ST QTN based on combined bioinformatics and haplotype analyses. The results provided useful germplasm and genetic information for future improvement of ST in rice.

## Electronic supplementary material

Supplementary Table S1, Supplementary Table S2, Supplementary Table S2, Supplementary Figure S1
