# Supplementary material for: Identification of QTN and candidate genes for Salinity Tolerance at the Germination and Seedling Stages in Rice by Genome-Wide Association Analyses
Source: Sci Rep. 2018 Apr 25;8:6505. doi: 10.1038/s41598-018-24946-3 (PMC5916932; doi:10.1038/s41598-018-24946-3)
Supplement: Supplementary file 1 — Supplementary Table S1, Supplementary Table S2, Supplementary Table S2, Supplementary Figure S1 [file 41598_2018_24946_MOESM1_ESM.pdf]

Identification of QTN and candidate genes for Salinity Tolerance at the Germination and Seedling Stages in Rice by Genome-Wide Association Analyses

Shahzad Amir Naveed[1], Fan Zhang[1], Jian Zhang[1], Tian-Qing Zheng[1], Li-Jun Meng[1,2], Yun-Long Pang[1], Jian-Long Xu[1,2*], Zhi-Kang Li[1,2*]

[1] Institute of Crop Sciences/National Key Facility for Crop Gene Resources and Genetic Improvement, Chinese Academy of Agricultural Sciences, 12 South Zhong-Guan-Cun Street, Beijing, China,

[2] Shenzhen Institute of Breeding and Innovation, Chinese Academy of Agricultural Sciences, Shenzhen, China

*Correspondence: Zhi-Kang Li (email: lizhikang@caas.cn), Jian-Long Xu (email: xujlcaas@126.com; Tel: 010-82105854)

Running Title: GWAS for Salt tolerance in Rice

Supplementary Table S1: ANOVA for the measured traits under salinity for 208 *indica* rice germplasms.

Trait	Source of	df	SS	MS	F	P- value	R^2 (%)
RL	Genotypes	207	214.262	1.035	26.975	<0.0001	87.3
	Rep.	1	0.002	0.002	0.050	0.823	
	Residuals	208	7.981	0.038			
	Total	415	222.245				
SL	Genotypes	207	161.477	0.780	54.508	<0.0001	95.2
	Rep.	1	0.092	0.092	6.451	0.012	
	Residuals	208	2.977	0.014			
	Total	415	164.546				
GR	Genotypes	207	196724.885	950.362	495.316	<0.0001	82.4
	Rep.	1	62.931	62.931	32.799	0.0001	
	Residuals	208	399.089	1.919			
	Total	415	197186.905				
RDW	Genotypes	207	0.040	0.000	50.706	<0.0001	86.1
	Rep.	1	0.000	0.000	1.065	0.303	
	Residuals	208	0.001	0.000			
	Total	415	0.041				
SDW	Genotypes	207	0.616	0.003	3.945	<0.0001	51.3
	Rep.	1	0.032	0.032	42.195	0.0001	
	Residuals	208	0.157	0.001			
	Total	415	0.804				
SST	Genotypes	207	792.632	3.829	10.966	<0.0001	79.3
	Rep.	1	0.868	0.868	2.485	0.116	
	Residuals	208	72.632	0.349			
	Total	415	866.132				
SSD	Genotypes	207	259.608	1.254	7.648	<0.0001	76.77
	Rep.	1	3.437	3.437	20.961	0.0001	
	Residuals	208	34.108	0.164			
	Total	415	297.154				
RNa	Genotypes	207	29.448	0.142	88.241	<0.0001	87.2
	Rep.	1	0.004	0.004	2.678	0.103	
	Residuals	208	0.335	0.002			
	Total	415	29.787				
SNa	Genotypes	207	148.107	0.715	80.852	<0.0001	85.2
	Rep.	1	0.023	0.023	2.626	0.107	
	Residuals	208	1.841	0.009			
	Total	415	149.971				
RKC	Genotypes	207	6.708	0.032	291.332	<0.0001	90.5

	Rep.	1	0.000	0.000	0.547	0.461	
	Residuals	208	0.023	0.000			
	Total	415	6.731				
SKC	Genotypes	207	9.273	0.045	89.813	<0.0001	92.3
	Rep.	1	0.001	0.001	1.990	0.160	
	Residuals	208	0.104	0.000			
	Total	415	9.378				
RNK	Genotypes	207	399.556	1.930	89.533	<0.0001	96.7
	Rep.	1	0.014	0.014	0.650	0.421	
	Residuals	208	4.484	0.022			
	Total	415	404.054				
SNK	Genotypes	207	198.896	0.961	50.557	<0.0001	95.3
	Rep.	1	0.031	0.031	1.625	0.204	
	Residuals	208	3.953	0.019			
	Total	415	202.880				

Supplementary Table S2: Allelic distribution of the 22 candidate genes for eight important QTL regions.

QTL	position	REF<->ALT	+/-	ID	CODEN	AAA	NonSyn
qSNK1	1-11460354	G<->K	-	*Os01g20160*	GAC<->GAA;	D<->E;	0.5
qSNK1	1-11462858	G<->S	-	*Os01g20160*	CCA<->GCA;	P<->A;	0.5
qSNK1	1-11527219	A<->R	+	*Os01g20720*	AAT<->AGT;	N<->S;	0.5
qSNK1	1-11528968	A<->R	+	*Os01g20720*	AAA<->AGA;	K<->R;	0.5
qSNK12	12-4046018	G<->R	+	*Os12g07970*	CGT<->CAT;	R<->H;	0.5
qSNK12	12-4046366	C<->Y	+	*Os12g07970*	ACT<->ATT;	T<->I;	0.5
qSNK12	12-4056582	G<->S	-	*Os12g07990*	CCT<->CGT;	P<->R;	0.5
qSNK12	12-4058485	C<->Y	-	*Os12g07990*	GTC<->ATC;	V<->I;	0.5
qSNK12	12-4083192	A<->R	+	*Os12g08030*	ACT<->GCT;	T<->A;	0.5
qSNK12	12-4089798	G<->R	-	*Os12g08040*	ACG<->ATG;	T<->M;	0.5
qSNK12	12-4089813	A<->R	-	*Os12g08040*	GTG<->GCG;	V<->A;	0.5
qSNK12	12-4089972	G<->R	-	*Os12g08040*	CCG<->CTG;	P<->L;	0.5
qSNK12	12-4090089	C<->M	-	*Os12g08040*	TGG<->TTG;	W<->L;	0.5
qSNC1	1-23776512	T<->Y	+	*Os01g41930*	ATG<->ACG;	M<->T;	0.5
qSNC1	1-23776967	T<->Y	+	*Os01g41930*	TTT<->CTT;	F<->L;	0.5
qSNC1	1-23845674	A<->W	+	*Os01g42040*	AAA<->AAT;	K<->N;	0.5
qSNC1	1-23845952	G<->R	+	*Os01g42040*	AGC<->AAC;	S<->N;	0.5
qSNC1	1-23846057	A<->R	+	*Os01g42040*	GAA<->GGA;	E<->G;	0.5
qSNC1	1-23846329	G<->K	+	*Os01g42040*	GAT<->TAT;	D<->Y;	0.5
qSNC6	6-17556991	T<->W	+	*Os06g30390*	CTA<->CAA;	L<->Q;	0.5
qSNC6	6-17559929	G<->S	+	*Os06g30390*	AGT<->ACT;	S<->T;	0.5
qSNC6	6-17587542	T<->Y	-	*Os06g30440*	AGC<->GGC;	S<->G;	0.5
qSNC6	6-17587551	T<->Y	-	*Os06g30440*	ACC<->GCC;	T<->A;	0.5
qSNC6	6-17588990	T<->K	-	*Os06g30440*	TAT<->TCT;	Y<->S;	0.5
qRNK2	2-24278786	C<->M	+	*Os02g40100*	GCG<->GAG;	A<->E;	0.5
qRNK2	2-24279269	C<->S	+	*Os02g40100*	GCT<->GGT;	A<->G;	0.5
qRNK2	2-24279275	A<->R	+	*Os02g40100*	AAT<->AGT;	N<->S;	0.5
qRNK2	2-24279370	A<->R	+	*Os02g40100*	ATA<->GTA;	I<->V;	0.5
qRNK2	2-24280080	C<->M	+	*Os02g40100*	CAT<->AAT;	H<->N;	0.5
qRNK2	2-24280353	A<->R	+	*Os02g40100*	ATG<->GTG;	M<->V;	0.5
qRNK2	2-24288919	A<->W	-	*Os02g40120*	TTT<->TAT;	F<->Y;	0.5
qRNK2	2-24289438	C<->S	-	*Os02g40120*	GTC<->CTC;	V<->L;	0.5
qRNK2	2-24289637	G<->R	-	*Os02g40120*	TCA<->TTA;	S<->L;	0.5
qRNK2	2-24316864	G<->R	+	*Os02g40180*	GTG<->ATG;	V<->M;	0.5
qRNK2	2-24317291	C<->Y	+	*Os02g40180*	ACC<->ATC;	T<->I;	0.5
qRNK2	2-24381277	T<->Y	-	*Os02g40270*	GAG<->GGG;	E<->G;	0.5
qRNK2	2-24381350	T<->Y	-	*Os02g40270*	ACT<->GCT;	T<->A;	0.5
qRNK2	2-24381432	T<->K	-	*Os02g40270*	GAA<->GAC;	E<->D;	0.5

qRNK2	2-24381530	A<->W	-	*Os02g40270*	TGC<->AGC;	C<->S;	0.5
qRNK2	2-24381665	G<->K	-	*Os02g40270*	AAC<->AAA;	N<->K;	0.5
qRNK2	2-24394913	A<->W	-	*Os02g40280*	TAT<->TAA;	Y<->U;	0.5
qRNK2	2-24395150	A<->W	-	*Os02g40280*	ATA<->AAA;	I<->K;	0.5
qRNK2	2-24395800	T<->Y	-	*Os02g40280*	CAA<->CGA;	Q<->R;	0.5
qSDW9a	9-1770943	A<->R	-	*Os09g03590*	TTC<->CTC;	F<->L;	0.5
qSDW9a	9-1771405	C<->Y	-	*Os09g03590*	GAG<->AAG;	E<->K;	0.5
qSDW9a	9-1771758	C<->Y	-	*Os09g03590*	GTG<->ATG;	V<->M;	0.5
qSDW9a	9-1772281	T<->K	-	*Os09g03590*	GAA<->GAC;	E<->D;	0.5
qSDW9a	9-1845864	A<->R	-	*Os09g03670*	TGC<->CGC;	C<->R;	0.5
qSDW9a	9-1846300	G<->S	-	*Os09g03670*	TTC<->TTG;	F<->L;	0.5
qSDW9a	9-1846370	C<->Y	-	*Os09g03670*	CGC<->CAC;	R<->H;	0.5
qSDW9a	9-1898693	C<->Y	+	*Os09g03750*	CCA<->CTA;	P<->L;	0.5
qSDW9a	9-1900233	A<->R	+	*Os09g03750*	TAT<->TGT;	Y<->C;	0.5
qSST5	5-7039970	C<->Y	+	*Os05g12270*	CGA<->TGA;	R<->U;	0.5
qSST5	5-7040229	C<->Y	+	*Os05g12270*	CCG<->CTG;	P<->L;	0.5
qSST5	5-7040334	G<->R	+	*Os05g12270*	TGG<->TAG;	W<->U;	0.5
qSST5	5-7056012	C<->Y	+	*Os05g12290*	TCG<->TTG;	S<->L;	0.5
qSST5	5-7056096	C<->Y	+	*Os05g12290*	GCG<->GTG;	A<->V;	0.5
qSST5	5-7056957	G<->R	+	*Os05g12290*	TGT<->TAT;	C<->Y;	0.5
qSST5	5-7057038	C<->Y	+	*Os05g12290*	TCG<->TTG;	S<->L;	0.5
qSST5	5-7057974	A<->R	+	*Os05g12290*	AAT<->GAT;	N<->D;	0.5
qSST5	5-7058304	C<->Y	+	*Os05g12290*	CAA<->TAA;	Q<->U;	0.5
qSST9	9-20727751	C<->S	-	*Os09g35970*	GTG<->CTG;	V<->L;	0.5
qGR3	3-6902394	A<->R	+	*Os03g12840*	GAC<->GGC;	D<->G;	0.5
qGR3	3-6906211	C<->Y	+	*Os03g12840*	CTC<->CCC;	L<->P;	0.5

Supplementary Table S3: Haplotype analysis of 22 candidate genes for eight important QTL regions.

QTL	*ID*	Haplotype	Genotypes	Trait mean	STD	
qSNK1	*Os01g20160*	**				
		CC	32	2.74	0.63	a
		CG	14	1.9	0.85	ab
		AG	22	1.21	0.5	b
	Os01g20720	**				
		AA	15	2.93	0.73	a
		AG	10	1.98	1.01	ab
		GG	48	1.3	0.6	b
qSNK12	*Os12g07970*					
		GC	57	1.95	0.87	
		AT	68	1.69	1.02	
	Os12g07990	**				
		CA	46	3.7	0.54	a
		GG	56	2.3	0.4	b
	Os12g08030	**				
		A	51	2.97	0.6	a
		G	57	1.2	0.5	b
	Os12g08040					
		TTCG	18	2.94	0.92	
		CTCG	25	1.98	0.65	
		CCTG	28	2.61	0.84	
		TCTT	29	2.15	0.75	
qSNC1	*Os01g41930*	**				
		TC	15	3.91	0.74	a
		TT	37	3.31	0.95	ab
		CC	22	3.24	1	ab
		CT	18	2.18	0.63	b
	Os01g42040					
		TGAT	28	2.87	0.94	
		TAAT	22	1.96	1.1	
		AAGG	15	2.72	0.71	
		AAAG	18	3.45	0.74	
qSNC6	*Os06g30390*	**				
		TC	22	2.71	0.53	a
		AG	38	2.18	0.97	ab
		AC	12	1.38	0.48	b
	Os06g30440					
		AAC	38	2.91	0.86	
		AGA	36	2.47	0.86	

QTL	ID	Haplotype	Genotypes	Trait mean	STD	
		GGC	32	1.96	0.95	
qRNK2	*Os02g40100*	**				
		CGGGAA	20	3.59	0.67	a
		CCAACG	12	2.58	0.97	ab
		AGGGAA	26	1.24	0.59	b
	Os02g40120	**				
		TGC	34	3.69	0.97	a
		ACC	30	2.87	1.01	ab
		ACT	16	1.54	0.61	b
	Os02g40180	**				
		AC	21	3.96	0.73	a
		GC	12	2.37	0.89	ab
		GT	31	2.13	0.54	b
	Os02g40270					
		GGAAC	31	2.53	0.78	
		GAATA	36	1.95	1.03	
		AGCTA	22	2.86	0.96	
		AACAC	38	2.16	0.71	
	Os02g40280	**				
		TCG	14	3.74	0.71	a
		AGA	26	3.14	0.94	ab
		AGG	57	2.1	0.54	b
qSDW9a	*Os09g03590*					
		GTGA	38	0.096	0.032	
		GCAA	40	0.089	0.016	
		CTAC	35	0.092	0.026	
		CCGA	46	0.088	0.027	
	Os09g03670					
		TCG	44	0.099	0.027	
		CCA	37	0.096	0.016	
		TGA	31	0.092	0.032	
		CGA	30	0.082	0.026	
	Os09g03750	**				
		CA	14	0.13	0.009	a
		TA	65	0.096	0.009	b
		TG	74	0.092	0.009	b
qSST5	*Os05g12270*					
		CCG	75	8.5	1	
		CTA	53	7	1.5	
		TTA	18	6	2	
	Os05g12290	**				

QTL	*ID*	Haplotype	Genotypes	Trait mean	STD	
		CCGCAC	32	8	1	a
		CTGCGT	45	6.5	1	ab
		TCATAT	25	5.5	1	b
qSST9	*Os09g35970*	*				
		G	68	8	1	a
		C	26	5.5	1	b
qGR3	*Os03g12840*	**				
		CT	32	50	9	a
		TC	36	30	8	b

*and **Represents significance at P < 0.05 and P < 0.01, respectively.

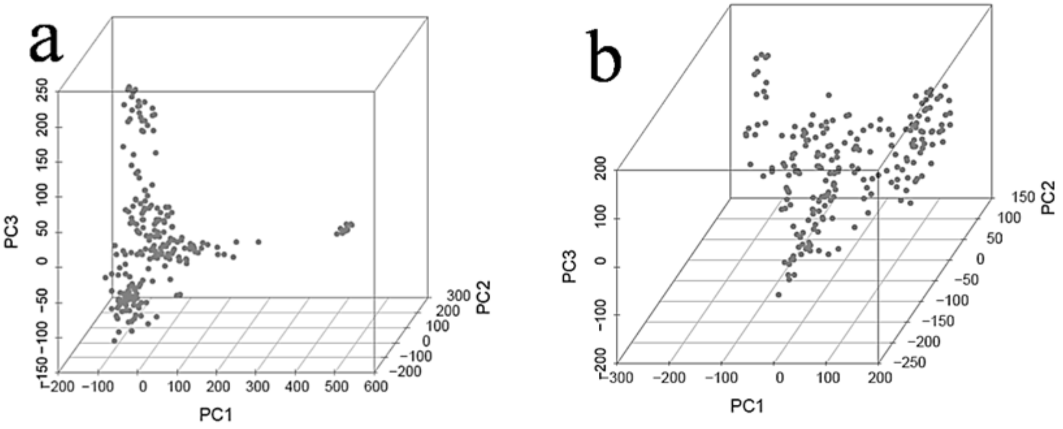

Supplementary Figure S1: PCA 3D plot of the first three principal components (PC) in 219 accessions (a) and 208 accessions (b), respectively.
